# 1036. Timing and Use of Adjunctive Steroids in Adults with Bacterial Meningitis: Compliance with International Guidelines.

**DOI:** 10.1093/ofid/ofac492.877

**Published:** 2022-12-15

**Authors:** Denisse Ramirez, Masayuki Nigo, Rodrigo Hasbun

**Affiliations:** UT Health Mc Govern Medical School, Houston, Texas; UT Health Mc Govern Medical School, Houston, Texas; UT Health Mc Govern Medical School, Houston, Texas

## Abstract

**Background:**

Adjunctive steroids decrease mortality in adults with bacterial meningitis with the exception of *Listeria monocytogenes*. Steroids given within 20 minutes, 4 hours and 12 hours after the first dose of antibiotic are advocated by the Infectious Diseases Society of America (IDSA), European, and United Kingdom (UK) guidelines, respectively. Compliance with these guidelines in the US is unknown.

**Methods:**

Retrospective observational study of 195 adults with community-acquired bacterial meningitis at 16 hospitals in Houston area from December 2004-May 2019.

**Results:**

Adjunctive steroids were given to 146/195 (75%) of patients and were more likely used in patients with a positive Gram stain, those with pneumococcal etiology, comorbidities, a higher CSF protein and with lower CSF glucose (P >0.05). The percentage of patients with pneumococcal etiology 77%. There was no association between the use of steroids and history of immunosuppression, fever, headache, stiff neck, Glasgow coma scale, seizures, focal neurological exam or serum WBC counts (P >0.05). Out of the 146 patients that received steroids, 28 (14%), 68 (35%), and 106 (54%) received them within 20 minutes, 4 hours or 12 hours after the first dose of antibiotics as per IDSA, European and UK guidelines, respectively. Use of steroids in this study by any timeline was not associated with improved clinical outcomes.

**Conclusion:**

Timing and use adjunctive steroids in adults with bacterial meningitis remain suboptimal in the US and could account for the lack of impact on clinical outcomes.

**Disclosures:**

**Rodrigo Hasbun, MD MPH FIDSA**, Biofire: Grant/Research Support|Biofire: Honoraria.

